# Volvulus of the Sigmoid Colon during Pregnancy: A Case Report

**DOI:** 10.1155/2012/641093

**Published:** 2012-02-09

**Authors:** Enzo Fabrício Ribeiro Nascimento, Michelle Chechter, Fábio Piovezan Fonte, Nara Puls, Juliana Santos Valenciano, Cláudio Luciano Penna Fernandes Filho, Ronaldo Nonose, Crhistiny Emmanuelle Gabriel Bonassa, Carlos Augusto Real Martinez

**Affiliations:** ^1^Department of General Surgery, São Francisco University Hospital, Bragança Paulista, SP, Brazil; ^2^São Francisco University Medical School, SP, Brazil; ^3^Division of General Surgery, São Francisco University Medical School, SP, Brazil; ^4^Postgraduate Program in Health Sciences, University of São Francisco, Rua José Raposo de Medeiros, 474, apto.602, 12914-450 Bragança Paulista, SP, Brazil

## Abstract

Colonic obstruction due to sigmoid colon volvulus during pregnancy is a rare but complication with significant maternal and fetal mortality. We describe a case of sigmoid volvulus in a patient with 33 weeks of gestation that developed complete necrosis of the left colon. *Case*. 27-year-old woman was admitted with 3 days of abdominal distention, vomit, and the stoppage of the passage of gases and feces. She was admitted with poor clinical conditions with septic shock, acute respiratory distress syndrome, and signs of diffuse peritonitis. Abdominal radiography showed severe dilation of the colon with horseshoe signal suggesting a sigmoid volvulus, pneumoperitoneum and we could not we could not identify fetal heartbeats. With a diagnosis of complicate sigmoid volvulus she was underwent to the laparotomy where we found necrosis of all descending colon due to double twist volvulus of the sigmoid. We performed a colectomy with a confection of a proximal colostomy, and closing of the rectal stump. Due to an uncontrollable uterine bleeding during cesarean due, it was required a hysterectomy. The patient had an uneventful postoperative course thereafter and was discharged on a regular diet on the 15th postoperative day.

## 1. Introduction

The diagnosis of complicated intestinal obstruction due to sigmoid volvulus (SV) during pregnancy is a rare clinical situation of extreme gravity, because the high rates of maternal and fetal mortality, especially if not recognized and treated precociously [1]. Since the initial report by Braun in 1885, it is estimated that less than 80 cases have been reported in the world literature; however, since 2005, only five cases have been reported [1–7]. Although the incidence of intestinal obstruction in the presence of pregnancy is not well defined, it is estimated that it can be achieved around a 1 : 1,500 to 1 : 66,431 birth cases [8, 9]. The causes of intestinal obstruction during pregnancy are similar to what occurs in their absence: adhesions, abdominal wall hernias, cancer of the left colon, internal hernias, Meckel's diverticulum, SV, and intussusceptions of sigmoid colon [7, 9].

The SV is the most frequent cause of intestinal obstruction during pregnancy accounting for 25% to 44% of published cases [10–12]. In endemic regions for Chagas disease, as South America, digestive manifestations are common and SV is a possible complication during pregnancy [13]. The main problem of SV in pregnancy is that of delay in presentation and diagnosis. Delay in diagnosis invariably leads to ischemia, necrosis, and perforation of the colon, and prompt surgical intervention is necessary to minimize the high rates of maternal and fetal mortality [9].

The purpose of this paper is to present a complicated case of intestinal obstruction, a consequence of SV, with double twist of the sigmoid mesocolon, in the 33rd week of gestation in a woman who did not have Chagas disease, which required urgent surgical treatment.

## 2. Case Report

A 27-year-old lady at 33 weeks of gestation, with a history of normal pregnancy and an abortion at 32 weeks' gestation without apparent cause. The patient did not have family history of complications of megacolon; pregnancy was going on with medical monitoring and had no abnormalities.

**Figure 1:**
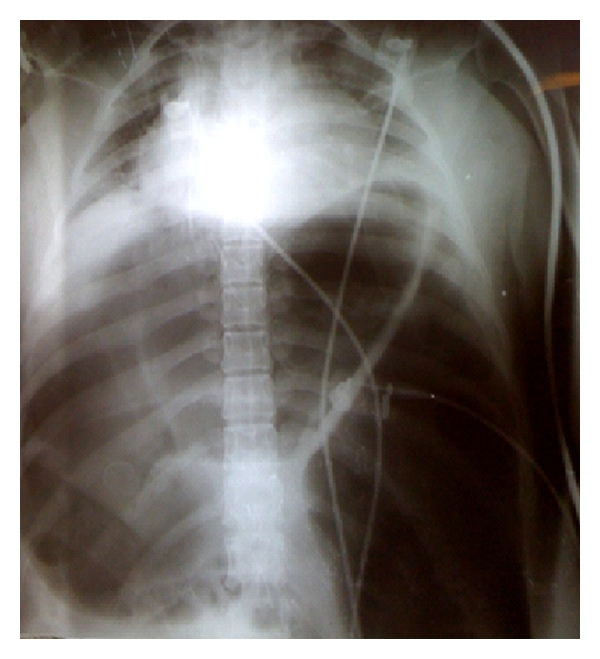
Plain radiography of the chest and abdomen showing severe distension of the transverse and sigmoid colon leading to a lung collapse.

She was admitted to the emergency room with complains of abdominal pain over the last three days, with progressive worsening despite the use of antispasmodics. On physical examination, the patient was confused, dehydrated, with severe respiratory distress, fever, reduced peripheral perfusion, and hypotension. The abdomen showed asymmetric distention more prominent on left flanks. Bowel sounds were absent. During the obstetric examination, it was not possible to detect fetal movements and fetal heart sounds were absent, suggesting intrauterine fetal death. On vaginal examination, the cervix was located in a mid-posterior position, without dilatation. Routine laboratory examination results were normal except for an elevated white blood cell count of 18.1 × 103/*μ*L. Abdominal radiographs revealed an abnormal gas pattern, with a dilated colon in the upper abdomen and air fluid levels. The colon showed significant dilation with the image in “horseshoe” suggesting the presence of SV. There were no signs of pneumoperitoneum. Chest radiography showed a significant elevation of the diaphragm, restricting pulmonary function (Figure 1). Ultrasonography of the abdomen and pelvis confirmed fetal death and showed the presence of moderate amounts of free fluid in the abdominal cavity.

**Figure 2:**
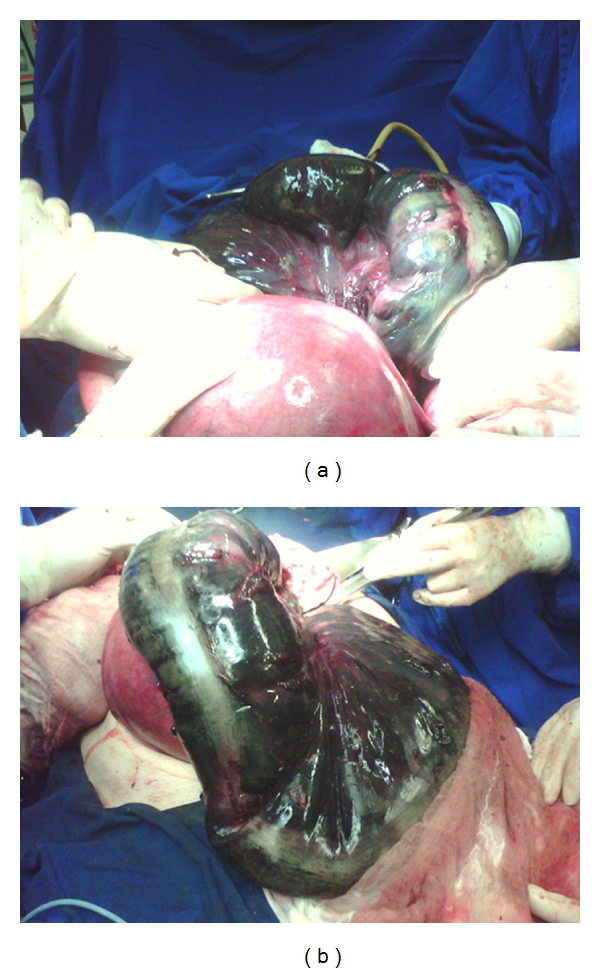
(a) Necrosis of the sigmoid colon after the pregnant uterus. Volvo, (b) complete necrosis of the sigmoid colon and descending portion of the transverse flow.

She was submitted to initial resuscitation with IV fluids, nasogastric suction, and bladder tube. The urinary catheter showed no urine output even after replacement of 2 L of saline. Despite having been kept since admission with oxygen mask, the patient developed an acute respiratory distress syndrome that required orotracheal intubation and mechanical ventilation. After initial resuscitation, with suspected bowel obstruction complicated by SV and intrauterine fetal death, the patient was taken emergently for exploratory laparotomy under general anesthesia. The abdominal cavity was accessed by laparotomy and after opening the peritoneum it was noted leaking from hemorrhagic, lots of foul smell and intense distension of loops of small intestine and colon. At laparotomy, an enormously distended sigmoid loop, with gangrenous changes extending from the transverse to extraperitoneal rectum, was found. The necrosis of the colon resulted from the presence of an SV, due to double twist on sigmoid mesocolon. The necrotic colon was posteriorly displaced by the pregnant uterus, without signs of perforation (Figures 2(a) and 2(b)). 

As there was no response to induction via vaginal labor and the patient conditions were critical, it was decided to carry out a concomitnt cesarean section. However, after removal of the dead fetus, the patient showed a uterine atony with severe bleeding, about 800 mL of blood, and it was refractory to standard clinical measures. Unfortunately, she required a concomitant total abdominal hysterectomy.

Faced with irreversible colon necrosis, resection of sigmoid colon and descending colon and proximal colostomy was performed. The closure of the rectal stump, below the peritoneal reflection, was performed with mechanical suture. As there was no response to induction via vaginal labor and the patient conditions were critical, it was decided to carry out a postmortem cesarean section. However, after removal of the dead fetus, the patient showed a uterine atony by severe bleeding, about 800 mL of blood, and it was refractory to standard clinical measures. Unfortunately, she required a concomitant total abdominal hysterectomy. After cesarean section, the peritoneal cavity was washing with saline and the abdominal wall was closing by planes. As the patient remained shocked and with respiratory distress syndrome during the surgical procedure, and she had received 4 UI of the plasma and 1 UI of the red blood cell concentration, the postoperative was followed at unit of intensive care. She remained for four days on mechanical ventilation and broad-spectrum antibiotic therapy. The renal and respiratory failure was improving gradually being transferred on the 7th day to the infirmary. With progressive improvement in general condition and normalization of pulmonary and renal function, she was discharged on 15th postoperative days. Colostomy was closed after three months and colorectal anastomosis was done.

## 3. Discussion

 SV usually occurs in institutionalized, debilitated, chronically patients who have long redundant sigmoid colon [11]. A high incidence has been reported in South America, due to high prevalence of chagasic megacolon, in Africa, and India, which has been attributed to the higher-fiber diet [13, 14]. Pregnancy increases the incidence of SV because the enlarging uterus can cause a redundant or abnormally long sigmoid colon [11, 15]. The occurrence of SV during pregnancy is considered an extremely rare and severe disease. A review of world medical literature revealed less than 80 cases of SV during pregnancy since the first case was reported [14–16].

The SV is the most common cause of intestinal obstruction during pregnancy, accounting for 25% to 44% of the cases and should therefore always be considered as one of the main possibilities of acute intestinal obstruction during pregnancy [2, 7, 12]. The mechanism of SV in pregnancy has been suggested to be due to displacement of an abnormally mobile sigmoid colon by the enlarging uterus that rise out of the pelvis and can twist around its fixation of point on the sigmoid mesocolon or the pelvic side wall [11, 15]. This could probably explain the increased incidence of SV in the third trimester [7, 9, 12]. Eight of the 13 recent cases published since 1983 were in the third trimester [5, 7].

The diagnosis of SV in pregnancy is often delayed because the symptoms mimic typical pregnancy-associated complaints [10]. The classical signs of bowel strangulation, such as vomiting, distention, and constipation, can be diminished or even absent during pregnancy [7, 17]. The most prevalent signs of obstruction were abdominal pain, asymmetric abdominal distension, and leukocytosis. In the initial phase, the abdominal pain was a mild colicky, but became constant and severe, probably due to vascular compromise [18]. The plain abdominal roentgenograms demonstrate typical patterns of obstruction in 80–91% of the cases, showing the characteristic “horseshoe” signal. In the patient here reported, the plain abdominal radiography could suspect of SV as a cause of intestinal obstruction. A detailed ultrasound examination may help in the differential diagnosis and confirm the presence of free fluids in the abdominal cavity, and confirm the viability of the fetus.

Diagnosis of the condition is often delayed. The average length of time from the onset of obstructive symptoms until presentation is reported to be 48 hours [9]. This is mainly because pregnancy itself clouds the clinical picture since abdominal pain, nausea, and leukocytosis can occur in an otherwise normal pregnancy [19]. The patient present in this report sought emergency medical care only after 72 hours of onset of symptoms, which may have contributed significantly to the delay in diagnosis and the necrosis of the twisted colon. Sometimes, the reluctance to obtain radiological evaluation in pregnancy may contribute to diagnostic delay and also could be responsible for the prohibitive maternal and fetal mortality. Early diagnosis of VS in pregnancy is still a great challenge and represents the most important factor to reduce the high rates of mortality. Since the time from beginning of symptoms to surgery is at least 48 hours, 100% of the cases had ischemia and necrosis and all needed a colonic resection [7]. In most cases the signs and symptoms allow the diagnosis of intestinal obstruction syndrome, though the correct diagnosis is usually findings performed during examination or surgery.

In South America, due to higher incidence of acquired megacolon, capable of causing SV, it becomes mandatory to search for personal or family history of Chagas disease, especially those pregnant women from endemic areas, with previous history of chronic intestinal constipation or other comorbidities associated with the disease (chagasic myocardiopathy and megaesophagus). The patient in this paper, despite being from a region where Chagas disease is still often diagnosed, showed negative immunoassay for disease. In countries where Chagas disease is rare, SV during the pregnancy can be found in chronically constipated patients, who often have redundant sigmoid colon. The patient described in this paper referred previous complaints of chronic intestinal constipation and occasionally she needed use of laxatives.

The management of SV in the pregnant patient involves aggressive fluid resuscitation, decompression of the proximal bowel, and recognition of this entity as an acute surgical emergency [9, 19–21]. In the absence of peritoneal signs or mucosal ischaemia, it would seem reasonable to attempt detorsion and decompression via sigmoidoscopic placement of a soft rectal tube, volvulus distortion through a flexible sigmoidoscopy, or colonoscopy until delivery of a viable infant [14, 15]. This approach can be repeated in recurrent cases until the second trimester when sigmoid colectomy is recommend. In cases where the disease is recurrent and could distort the SV by endoscopy, elective sigmoidectomy could be performed safely in the second trimester of pregnancy, reducing the chance of developing new twist during the course of the pregnancy [22]. However, in order for this strategy to be indicated, it is an essential prerequisite to prevent the existence of irreversible ischemia in the twisted segment of the sigmoid colon, which is not always easy to be confirmed by endoscopic examination [1].

 It is technically difficult to operate in the pelvis in the third trimester. In this period of pregnancy, when there is no ischemic necrosis of SV, it is also possible to indicate the endoscopic treatment deferring to sigmoidectomy after delivery, in order to preserve the fetus. Hence it is acceptable to do colonoscopic detorsion and tube decompression until fetal maturity, when elective labour followed by sigmoidectomy would provide a definitive treatment. Although colonoscopic detorsion is often successful in nonpregnant patients, successful use of this approach in late pregnancy is rarely reported [14, 15]. This could probably be due to the large gravid uterus acting as a mechanical impediment to detorsion. When there is fetal maturity, it can be performed at cesarean section and subsequent sigmoid fixation. With the advent of laparoscopy access, the option by sigmoidectomy or sigmoid fixation has been recommended during pregnancy or after childbirth [6].

When intestinal obstruction complicates pregnancy, both mother and fetus are at risk [1, 10]. Even employing an aggressive risk of maternal-fetal death is not negligible. Studies have shown that in a group of women who had intestinal obstruction, 23% required bowel resection for necrosis of the colon with a fetal mortality rate of 26% and 21% of maternal [9, 11]. Under these conditions, only early diagnosis and surgical indication as soon as possible can reduce these high indices. Although the complicated surgical treatment of SV has as main objective to reduce the high rates of maternal and fetal mortality, the optimal management to be taken is a particularly challenging situation [1, 21]. When an emergency surgical intervention is required in patients with SV complications, a standard midline incision allows maximal exposure with minimal uterine manipulation [5]. The ischaemic or necrotic bowel is resected with a diverting colostomy performed, with the stomata being sited away from an elective area of a possible cesarean section as the patient of the present paper [19, 21, 23, 24]. In most cases, the surgeon prefers resecting all necrotic bowel, exteriorizing the proximal colon as a terminal colostomy, and closing the distal rectum (Hartmann's procedure) [10, 23, 24]. Others prefer to perform a primary anastomosis with or without colonic cleansing intraoperatively when there is no contamination of the peritoneal cavity [10, 21]. Early diagnosis would make resection and primary anastomosis a safe approach, with the distinct advantage of reduced hospital stay and avoidance of further surgery. However, primary anastomosis of an unprepared distended paretic and oedematous colon is generally avoided as it could be hazardous to both mother and fetus [19].

There are also doubts about the best strategy on the fetus in cases of complicated SV. When the fetus is alive, the surgeon should try in every way to preserve the integrity of the uterus [16]. When there is fetal maturity and the patient is in stable condition, it can be induced a vaginal labor [16]. If it is not possible, and the pregnant woman is in satisfactory clinical conditions and the abdominal cavity has not signs of ischemic complications, it is possible to perform a cesarean section first, and then resection of nonviable intestinal segment, taking care to avoid disrupting the colon and consequent contamination of the uterine cavity.

When fetal death has occurred, some authors propose the implementation of cesarean section even with the previous diagnosis of fetal death [20, 24]. However, to use this strategy is prudent to initiate the procedure for removing the dead fetus and ensure that there is no contamination of the peritoneal cavity to avoid severe puerperal infection, which presents high rates of mortality [20, 24]. These arguments have recently been reinforced by a study showing that 57.9% of patients with failure of multiple organs and systems that have survived after being subjected to emergency abdominal surgery developed severe postpartum hemorrhage with 67.7% mortality puerperal sepsis, a situation similar to the case presented here [22].

In the patient of this paper, the presence of enlarged uterus made it difficult to assess the viability of the distal sigmoid colon, and especially high extraperitoneal rectum. It was possible to see that the area of necrosis of the sigmoid colon extended below to the peritoneal reflection; however, the conditions of rectum viability were difficult to assess. Since it was not possible to induce labor by vaginal delivery before. the laparotomy, due to the patient's own medical conditions, as this conduct would cause delay on surgery, despite the fetal death, it was chosen to performe the cesarean section, remove the dead fetus, and facilitate access to the retro-uterine space. After cesarean, the opening of the peritoneal reflection showed that necrosis extended to the upper portion of the extraperitoneal rectum making this segment needed to be resected together with the sigmoid colon. The patient remained in hypovolemic/septic shock during the procedure and due to her serious medical conditions, it was deemed reckless to reestablish colonic traffic not only by the heamodinamic instability but because the colon was not cleaned and the anastomosis would be located below the peritoneal reflection. The intraoperative colon cleansing to perform a primary anastomosis was also discarded because it could delay the operative time length. Therefore, we opted for resection of the necrotic colon and upper portion of the rectum, preparation of a proximal stoma, and closing the rectal stump, below the peritoneal reflection, by means of mechanical linear suture. As necrosis was compromising and extended from the rectum up to the middle portion of the transverse colon, the stoma was made on the right side of the abdomen.

Due to critical clinical conditions was not possible induce labor vaginally of a death fetus before the abdominal procedure. After complete removal of the necrotic bowel, it was decided to perform a caesarean section to remove the dead fetus. Unfortunately, the patient had severe uterine atony, with large blood loss refractory to conservative measures, being necessary concomitant hysterectomy. After surgery, the patient developed acute respiratory distress syndrome and renal failure requiring advanced support in the intensive care unit. She gradually recovered and was discharged on the 14th postoperative day.

SV should be considered when examining severe abdominal pain in a pregnant woman with a history of severe constipation. Early suspicion together with prompt intervention will minimize maternal and fetal morbidity and mortality of this rare complication of pregnancy [1].

## 4. Conclusion

 SV complicating pregnancy is an uncommon and potentially devastating development and should be recognized as a surgical emergency. Diagnosis requires a high index of suspicion in a patient who presents with complaints of abdominal pain and evidence of bowel obstruction. Delay in diagnosis and treatment beyond 48 hours results in colonic necrosis and increased fetal and maternal morbidity and mortality. Prompt intervention is necessary to minimize these complications and achieve a definitive cure.

## References

[1] Iwamoto I, Miwa K, Fujino T, Douchi T (2007). Perforated colon volvulus coiling around the uterus in a pregnant woman with a history of severe constipation. *Journal of Obstetrics and Gynaecology Research*.

[2] De U, De KK (2005). Sigmoid volvulus complicating pregnancy. *Indian Journal of Medical Sciences*.

[3] Narjis Y, El Mansouri MN, Jgounni R (2008). Sigmoid volvulus, a rare complication of pregnancy. *Gynecologie Obstetrique Fertilite*.

[4] Vo TM, Gyaneshwar R, Mayer C (2008). Concurrent sigmoid volvulus and herniation through broad ligament defect during pregnancy: case report and literature review. *Journal of Obstetrics and Gynaecology Research*.

[5] Machado NO, Machado L (2009). Sigmoid volvulus complicating pregnancy managed by resection and primary anastomosis: case report with literature review. *Sultan Qaboos University Medical Sciences Journal*.

[6] Azuar AS, Bouillet-Dejou L, Jardon K (2009). Laparoscopy during pregnancy: experience of the French university hospital of Clermont-Ferrand. *Gynecologie Obstetrique Fertilite*.

[7] Kolusari A, Kurdoglu M, Adali E, Yildizhan R, Sahin HG, Kotan C (2009). Sigmoid volvulus in pregnancy and puerperium: a case series. *Cases Journal*.

[8] Coughlan BM, O’Herlihy C (1978). Acute intestinal obstruction during pregnancy. *Journal of the Royal College of Surgeons of Edinburgh*.

[9] Perdue PW, Johnson HW, Stafford PW (1992). Intestinal obstruction complicating pregnancy. *American Journal of Surgery*.

[10] Redlich A, Rickes S, Costa SD, Wolff S (2007). Small bowel obstruction in pregnancy. *Archives of Gynecology and Obstetrics*.

[11] Ballantyne GH, Brandner MD, Beart RW, Ilstrup DM (1985). Volvulus of the colon. Incidence and mortality. *Annals of Surgery*.

[12] Connolly MM, Unti JA, Nora PF (1995). Bowel obstruction in pregnancy. *Surgical Clinics of North America*.

[13] Gabriel AG, Gabriel Neto S, Oliveira EC, Luquetti AO, Cleva R, Zilberstein B (2005). Gastric and transverse colonic volvulus in patient with chagasic megagastria and megacolon. *Arquivos Brasileiros de Cirurgia Digestiva*.

[14] Allen JC (1990). Sigmoid volvulus in pregnancy. *Journal of the Royal Army Medical Corps*.

[15] Alshawi JS (2005). Recurrent sigmoid volvulus in pregnancy: report of a case and review of the literature. *Diseases of the Colon and Rectum*.

[16] Fraser JL, Eckert LA (1983). Volvulus complicating pregnancy. *Canadian Medical Association journal*.

[17] Kusnetzoff DJ, Barata AD, Casalnuovo C, Alvarez LM (1997). Massive midgut volvulus during pregnancy. *Journal of Obstetrics and Gynaecology*.

[18] Ventura-Braswell AM, Satin AJ, Higby K (1998). Delayed diagnosis of bowel infarction secondary to maternal midgut volvulus at term. *Obstetrics and Gynecology*.

[19] Keating JP, Jackson DS (1985). Sigmoid volvulus in late pregnancy. *Journal of the Royal Army Medical Corps*.

[20] Lord SA, Boswell WC, Hungerpiller JC (1996). Sigmoid volvulus in pregnancy. *American Surgeon*.

[21] Safioleas M, Chatziconstantinou C, Felekouras E (2007). Clinical considerations and therapeutic strategy for sigmoid volvulus in the elderly: a study of 33 cases. *World Journal of Gastroenterology*.

[22] Pérez Assef A, Acevedo Rodríguez O, Del Consuelo Tamayo Gómez F, Oviedo Rodríguez R (2010). Characterization of obstetric patients with multiple organ failure in the intensive care unit of a Havana Teaching Hospital, 1998 to 2006. *MEDICC Review*.

[23] Mirza MS, Mulla M, Hall RI (2009). Large bowel obstruction in pregnancy: a rare entity, an unusual cause. *Archives of Gynecology and Obstetrics*.

[24] Joshi MA, Balsarkar D, Avasare N (1999). Gangrenous sigmoid volvulus in a pregnant woman. *Tropical Gastroenterology*.

